# Announcement of WormAtlas partnership with the Journal of Nematology

**DOI:** 10.21307/jofnem-2021-090

**Published:** 2021-11-03

**Authors:** Nathan E. Schroeder, David H. Hall

**Affiliations:** 1 Department of Crop Sciences, University of Illinois at Urbana-Champaign, Urbana, IL 61801; 2 Dominick P. Purpura Department of Neuroscience, Albert Einstein College of Medicine, Bronx, NY 14061

**Keywords:** *Heterodera* spp, *Meloidogyne* spp, Morphology, Plant-parasitic nematodes, *Pristionchus pacificus*, *Strongyloides* spp

## Abstract

A detailed understanding of nematode anatomy can be leveraged for the development of new parasitic nematode control strategies and for fundamental biological insights through nematode model organisms. The Center for *C. elegans* Anatomy, with its websites WormAtlas and WormImage, is the central anatomical resource for researchers studying the model organism *Caenorhabditis elegans*. Here, we announce our expansion of the WormAtlas and WormImage resources beyond *C. elegans* to include additional nematode species. Towards this goal, we will partner with the Journal of Nematology to write and solicit anatomically focused review chapters for publication in the Journal and corresponding inclusion on the WormAtlas website.

Anatomical maps are useful for predicting the function of tissues and for understanding evolutionary relationships among species. One reason for the success of *Caenorhabditis elegans* as a model organism is the beautiful serial-section electron microscopy (EM) data collected by Nichol Thomson and colleagues at the Medical Research Council during the 1970s and 80s (Perkins et al., 1986; Sulston et al., 1980, 1983; Ward et al., 1975; White et al., 1976, 1986). These data provided the basis for the first complete anatomical parts list of any animal species. Reconstruction of *C. elegans* anatomy has allowed researchers to conduct detailed mechanistic studies on the genetics of development and the function of individual cells within the context of a whole animal.

The NIH-funded Center for *C. elegans* Anatomy has served as a research resource for the *C. elegans* community since 1998. The Center developed and maintains the WormAtlas and WormImage websites. The handbook portion of WormAtlas includes thorough reviews of *C. elegans* anatomy organized by tissue type. The associated database, WormImage, hosts tens of thousands of *C. elegans* electron micrographs searchable by sex, genotype, developmental stage, and tissue. Due to technological limits at the time, most of the data associated with publications were never available to the public. We have digitized *C. elegans* micrograph collections from several laboratories, including the original EM data prepared by Nichol Thomson, and have these available with metadata on the WormImage website. Another resource, Slidable Worm, provides an interactive and annotated series of EM images through a wild-type adult hermaphrodite. Descriptions of all 302 hermaphrodite and 385 male neurons, anatomical methods, and quick links to key anatomical publications are also included as part of the resources in WormAtlas. Illustrations of individual cells are commonly used by researchers for presentations and publications.

While all nematodes have a relatively similar body-plan, *C. elegans* represents only one of the tens of thousands of anatomical variations in the phylum. During the next few years, we will expand WormAtlas beyond *C. elegans* to include descriptions and unpublished data from additional nematode species. We plan to solicit new review chapters on anatomy from experts in these species and encourage readers to contact us with recommendations. WormAtlas authors can leverage the expertise of the current WormAtlas team, including illustration services. As part of this change, we are partnering with the Journal of Nematology (JON) to submit these chapters for publication. WormAtlas reviews will be submitted through the established JON publishing site and undergo an identical peer-review process to other JON review articles. To demonstrate this new partnership, this issue of JON includes the newly completed chapter Introduction to *Pristionchus pacificus* Anatomy. Additional reviews are being solicited for other aspects of *P. pacificus* anatomy including the nervous system, stoma, and reproductive system.

Alongside these new chapters in WormAtlas, we plan to expand WormImage to include previously unpublished EM data from other species. To date, we have digitized about half of the large collection of original EM data collected by Dr. Burton Endo during his 30 year career (Endo and Wergin, 1977; Endo, 1980, 1983, 1984; Endo et al., 1997; Endo and Trpis, 1997; Wergin and Endo, 1976). These data comprise approximately 40,000 micrographs covering eight different species. Within this collection we have found micrographs from the plant-parasitic *Trichodorus similis,* which have never been included in any publications. Once annotated, these digitized data sets will be uploaded to the WormImage archive. We plan to add micrographs from additional collections as they become available.
